# Peptide-Dependent Recognition of HLA-B*57:01 by KIR3DS1

**DOI:** 10.1128/JVI.03586-14

**Published:** 2015-03-04

**Authors:** Geraldine M. O'Connor, Julian P. Vivian, Emma Gostick, Phillip Pymm, Bernard A. P. Lafont, David A. Price, Jamie Rossjohn, Andrew G. Brooks, Daniel W. McVicar

**Affiliations:** a Cancer and Inflammation Program, Center for Cancer Research, National Cancer Institute, Frederick, Maryland, USA; b Department of Biochemistry and Molecular Biology, School of Biomedical Sciences, Monash University, Clayton, Victoria, Australia; c Australian Research Council Centre of Excellence for Advanced Molecular Imaging, Monash University, Clayton, Victoria, Australia; d Institute of Infection and Immunity, Cardiff University School of Medicine, Heath Park, Cardiff, Wales, United Kingdom; e Non-Human Primate Immunogenetics and Cellular Immunology Unit, Laboratory of Molecular Microbiology, National Institute of Allergy and Infectious Diseases, National Institutes of Health, Bethesda, Maryland, USA; f Human Immunology Section, Vaccine Research Center, National Institute of Allergy and Infectious Diseases, National Institutes of Health, Bethesda, Maryland, USA; g Department of Microbiology and Immunology, University of Melbourne, Parkville, Victoria, Australia

## Abstract

Killer cell immunoglobulin-like receptors (KIRs) play an important role in the activation of natural killer (NK) cells, which in turn contribute to the effective immune control of many viral infections. In the context of HIV infection, the closely related KIR3DL1 and KIR3DS1 molecules, in particular, have been associated with disease outcome. Inhibitory signals via KIR3DL1 are disrupted by downregulation of HLA class I ligands on the infected cell surface and can also be impacted by changes in the presented peptide repertoire. In contrast, the activatory ligands for KIR3DS1 remain obscure. We used a structure-driven approach to define the characteristics of HLA class I-restricted peptides that interact with KIR3DL1 and KIR3DS1. In the case of HLA-B*57:01, we used this knowledge to identify bona fide HIV-derived peptide epitopes with similar properties. Two such peptides facilitated productive interactions between HLA-B*57:01 and KIR3DS1. These data reveal the presence of KIR3DS1 ligands within the HIV-specific peptide repertoire presented by a protective HLA class I allotype, thereby enhancing our mechanistic understanding of the processes that enable NK cells to impact disease outcome.

**IMPORTANCE** Natural killer (NK) cells are implicated as determinants of immune control in many viral infections, but the precise molecular mechanisms that initiate and control these responses are unclear. The activating receptor KIR3DS1 in combination with HLA-Bw4 has been associated with better outcomes in HIV infection. However, evidence of a direct interaction between these molecules is lacking. In this study, we demonstrate that KIR3DS1 recognition of HLA-Bw4 is peptide dependent. We also identify HIV-derived peptide epitopes presented by the protective HLA-B*57:01 allotype that facilitate productive interactions with KIR3DS1. Collectively, these findings suggest a mechanism whereby changes in the peptide repertoire associated with viral infection provide a trigger for KIR3DS1 engagement and NK cell activation.

## INTRODUCTION

The role of natural killer (NK) cells and of members of the killer cell immunoglobulin-like receptor (KIR) family in the control of viral infections is supported by a growing body of evidence from functional analyses and disease association studies. Particular KIRs have been implicated in the immune response to several persistent viruses, including human cytomegalovirus (HCMV), hepatitis C virus (HCV), human papillomavirus (HPV), and human immunodeficiency virus (HIV) (reviewed in reference 1). In the context of HIV, specific KIR genes, KIR/HLA combinations, and/or variations in KIR gene copy numbers have been linked with resistance to infection (2, 3), disease progression (4–6), and the development of opportunistic infections (7). In addition, functional experiments have demonstrated KIR/HLA-dependent NK cell expansion and cytotoxicity in relation to the control of viral replication (8–10). Nonetheless, the mechanistic basis for these observations remains obscure.

Members of the KIR family include both activating and inhibitory receptors expressed on the surface of NK cells and various T cell subsets (reviewed in reference 11). In each case, ligand recognition is mediated by either two (2D) or three (3D) extracellular Ig domains. Inhibitory KIRs possess a long (L) cytoplasmic tail containing immunoreceptor tyrosine-based inhibitory motifs (ITIMs) responsible for the transduction of a negative signal via recruitment of protein tyrosine phosphatases. In contrast, activating KIRs harbor a charged residue in the transmembrane domain together with a short cytoplasmic tail (S) and couple to the signaling adaptor DAP12.

The best-described KIR ligands are HLA class I molecules. KIR binding is focused on the α1 and α2 domains of the HLA molecule, and position 80 of the heavy chain has been shown to be a key specificity determinant for multiple KIRs (12–14). KIR3DL1 binds specifically to HLA-A and HLA-B molecules that possess the Bw4 public epitope (15). These interactions are modulated by the presented peptide, most notably via specific residues at the C terminus (12, 16). Consequently, NK cells can be sensitive to changes in the peptide repertoire even when HLA expression levels are maintained. In contrast, the role of activating KIRs is less well understood. Although several activating KIRs are very similar at the sequence level to their inhibitory counterparts (e.g., 2DL1/2DS1 and 3DL1/3DS1), evidence of HLA binding has been much more difficult to detect. For example, biochemical and functional analyses have shown that KIR2DS1 binds to HLA-C2 complexes with affinities that lie well below those observed for KIR2DL1 (17). This reduced HLA binding has been attributed to single KIR-specific amino acid polymorphisms (18–20), which appear to leave peptide preferences largely intact (17).

KIR3DS1 is the activating counterpart of KIR3DL1, exhibiting 97% amino acid similarity in extracellular domain. In a study of HIV-infected individuals, Martin et al. demonstrated that carriage of the *KIR3DS1* gene in conjunction with a subgroup of HLA-Bw4 (Bw4 80I) alleles was associated with slower progression to AIDS (21). Subsequent functional experiments revealed that NK cells from individuals with this compound genotype displayed higher levels of degranulation and were able to control HIV replication in autologous CD4^+^ T cells (8). Analysis of KIR3DS1 expression in HIV-infected patients further showed that KIR3DS1^+^ NK cell subsets expand during acute infection, most markedly in individuals with HLA-Bw4 80I (9). These results suggest an important combined role for KIR3DS1 and HLA-Bw4 80I in the immune response to HIV infection. However, in the absence of direct evidence for an interaction between KIR3DS1 and Bw4 80I allotypes, a mechanistic basis for genetic association has been lacking (8, 22, 23). Analysis of the amino acid differences between KIR3DL1 and KIR3DS1 reveals four key residues that may critically govern the differential HLA-Bw4 binding patterns of these closely related receptors; in each case, the KIR3DS1-like variant has been individually implicated as a determinant of reduced affinity for HLA (12, 24, 25). In contrast, a single amino acid change is responsible for the impaired HLA affinity of 2DS KIRs. It therefore seems feasible that KIR3DS1 may display unique specificities, either in terms of HLA recognition and/or peptide tolerance.

It is established that KIR interactions with HLA class I molecules are sensitive to changes in the presented peptide, especially at the C terminus (12). The ability to discriminate between peptides can also control NK cell activity (26). In this study, we used a structure-driven approach to characterize specific peptide preferences and identify HLA-Bw4-restricted ligands recognized by KIR3DS1.

## MATERIALS AND METHODS

### Cell lines.

HEK293T cells were maintained in Dulbecco's modified Eagle's medium (DMEM) supplemented with 10% fetal calf serum (FCS), 2 mM l-glutamine, 50 U/ml penicillin, and 50 μg/ml streptomycin. Jurkat cells stably expressing chimeric KIR3DS1-CD3ζ reporter constructs (22) were maintained in RPMI medium supplemented with 10% FCS, 2 mM l-glutamine, 50 U/ml penicillin, 50 μg/ml streptomycin, and 0.5 mg/ml Geneticin. Ba/F3 cells were maintained in RPMI medium supplemented with 10% FCS, 2 mM l-glutamine, 50 U/ml penicillin, 50 μg/ml streptomycin, and 10 ng/ml murine interleukin-3 (mIL-3).

### Mutagenesis and transfection studies.

KIR3DL1 and KIR3DS1 constructs with a 5′ FLAG tag sequence (GACTACAAAGACGATGACGACAAG) were cloned into a pEF6 vector. Specific nucleotide residues were mutated using a QuikChange II site-directed mutagenesis kit (Stratagene) with PAGE-purified primers and verified by direct sequencing. These constructs were introduced into HEK293T cells using FuGene6 transfection reagent (Roche) according to the manufacturer's instructions.

### Tetramer staining.

Fluorochrome-conjugated peptide-HLA (pHLA) class I tetramers (detailed in Table 1) were produced as described previously (27). Cells were stained at 48 h posttransfection with an optimal concentration of tetramer (0.2 μg with respect to the monomeric component in minimal residual volume) or anti-FLAG (clone M2; Sigma-Aldrich) monoclonal antibody (MAb) for 30 min at 4°C. In all cases, mock-transfected HEK293T cells were stained in parallel to identify any background staining. Ba/F3 cells stably expressing LILRB1 were used as a positive control for tetramer fidelity. For blocking experiments, cells were preincubated with the indicated MAb (10 μg/ml) for 15 min at 4°C.

**TABLE 1:** The peptide sequences and HLA molecules used to generate pHLA complexes in this study

Name	HLA	Peptide	Peptide source
LF9	B*57:01	LSSPVTKSF	Human
LF9 A8	B*57:01	LSSPVTKAF	Modified from human
LF9 E8	B*57:01	LSSPVTKEF	Modified from human
LF9 F8	B*57:01	LSSPVTKFF	Modified from human
LF9 H8	B*57:01	LSSPVTKHF	Modified from human
LF9 L8	B*57:01	LSSPVTKLF	Modified from human
LF9 R8	B*57:01	LSSPVTKRF	Modified from human
TW10	B*57:01	TSTLQEQIGW	HIV
TW10 G9D	B*57:01	TSTLQEQIDW	HIV
RW8	A*24:02	RYPLTFGW	HIV
RW8 W8A	A*24:02	RYPLTFGA	HIV
RW8 Y2F	A*24:02	RFPLTFAW	HIV
IF9	B*57:01	ISGKAKGWF	HIV
KY10	B*57:01	KAVRIKLFLY	HIV
AL9	B*57:01	AAFDLSFFL	HIV
ISY	B*57:01	ISYIMLIFF	Yellow fever virus
AW9	B*57:01	AAVKAACWW	HIV
KF9	B*57:01	KAAFDLSFF	HIV
RW9	B*57:01	RTIQGQRFW	Bacillus anthracis
YF9	B*57:01	YPASLHKFF	Marburg virus

### Reporter assays.

Jurkat cells stably expressing chimeric KIR3DS1-CD3ζ reporter constructs (22) were transiently transfected with an NFAT (nuclear factor of activated T cells)-luciferase plasmid by electroporation and then stimulated with plate-bound HLA class I monomer for 18 h at 37°C. Luciferase activity was measured after cell lysis using a Dual-Glo luciferase assay (Promega).

### Crystallization and data collection.

The HLA class I heavy chain and β2-microglobulin were refolded from inclusion body preparations expressed in Escherichia coli and purified as detailed previously (28). KIR3DL1*001 was expressed in HEK293S cells and purified from the secreted fraction by nickel affinity and size exclusion chromatography. The HLA-B*57:01-LF9.A8 (where LF9.A8 indicates the nine-residue peptide beginning with an L residue and ending with an F residue and with a substitution of an A residue at position 8, i.e., LSSPVTKAF) and HLA-B*57:01-LF9.E8 (LSSPVTKEF) binary complexes were concentrated to ∼12 mg/ml in 10 mM Tris, pH 8.0. The ternary KIR3DL1*001-HLA-B*57:01-LF9.A8 complex was concentrated to ∼10 mg/ml in 10 mM Tris, pH 8.0, and 300 mM NaCl. Crystals were obtained at 294 K by the hanging-drop vapor diffusion method. Binary complexes were crystallized from a reservoir solution comprising 28% polyethylene glycol (PEG) 8000, 0.2 M ammonium sulfate, and 0.1 M cacodylate, pH 6.25 (29). The ternary complex was crystallized from a reservoir solution comprising 16% PEG 3350, 0.1 M tri-sodium citrate, pH 6.0, and 4% Tacsimate, pH 5.0 (12). Prior to data collection, binary crystals were equilibrated in a crystallization solution with 10% glycerol added as a cryoprotectant and then flash cooled in a stream of liquid nitrogen at 100 K. The ternary crystals were similarly flash cooled in a cryoprotectant composed of reservoir solution supplemented with 35% PEG 3350. X-ray diffraction data were recorded on a Quantum-315 charge-coupled device (CCD) detector at the MX2 beamline of the Australian Synchrotron. Data were integrated and scaled using MOSFLM and SCALA from the CCP4 program suite (30). Details of the data processing statistics are provided in Table 2.

**TABLE 2:** Data collection and refinement statistics for the HLA-B*57:01-LF9.E8 binary and KIR3DL1*001-HLA-B*57:01-LF9.A8 ternary complexes

Parameter	Value(s) for:^*a*^	
	HLA-B*57:01-LF9.E8	KIR3DL1*001- HLA-B*57:01-LF9.A8
Data collection statistics		
Temp (K)	100	100
X-ray source	MX2 Australian synchrotron	MX2 Australian synchrotron
Space group	P2_1_	P1
Cell dimensions (Å)		
*a*, *b*, *c* (Å)	65.0, 49.3, 70.0	51.9, 62.0, 65.9
α, β, γ (°)	102.7^*e*^	94.6, 98.6,109.2
Resolution (Å)	40.0–1.90 (2.00–1.90)	40.0–2.30 (2.42–2.30)
Total no. of observations	126,910 (16,154)	107,830 (14,691)
No. of unique observations	33,403 (4,631)	29,881 (4,161)
Multiplicity	3.8 (3.5)	3.6 (3.5)
Completeness (%)	97.2 (93.5)	90.6 (84.7)
*I*/σ*_I_*	9.3 (1.8)	8.0 (2.5)
*R*_merge_^*b*^	0.09 (0.82)	0.10 (0.41)
Refinement statistics		
No. of nonhydrogen atoms		
Protein	3,105	5,281
Water	265	237
*R*_factor_^*c*^	0.206	0.216
*R*_free_^*c*^	0.247	0.257
RMSD from ideality^*d*^		
Bond length (Å)	0.010	0.004
Bond angle (°)	1.09	1.01
Ramachandran plot (%)		
Favored	96.0	97.2
Allowed	4.0	2.8
B factors (Å^2^)		
Avg main chain	28.9	38.2
Avg side chain	35.4	40.2
Avg water	39.8	39.4

a Values in parentheses are for the highest-resolution shell.

b *R*_merge_ = Σ*_hkl_* Σ*_j_* |*I_hklj_* − <*I_hkl_*> |/Σ*_hkl_* Σ*_j_ I*_hklj_, where *I_hklj_* is the *j*th observation of reflection *hkl*, <*I*> is the mean intensity of all observations of the reflection *hkl*, Σ*_hkl_* is taken over all reflections, and Σ*_j_* is taken over all observations of each reflection.

c *R*_factor_ = Σ*_hkl_* ||*F_o_*| − |*F_c_*||/Σ*_hkl_* |*F_o_*|: for all data excluding the 5% that comprised the *R*_free_ used for cross-validation, where *F_o_* are amplitudes of the structure factors for the observed data and *F_c_* are amplitudes of the structure factors calculated from the atomic model. The summation is carried over all data points *hkl* included in the model derivation.

d RMSD, root mean square deviation.

e Value is for β.

### Structure determination and refinement.

Structures were determined by molecular replacement as implemented in Phaser (31). The search model used for the ternary complex was the previously determined structure of KIR3DL1*001-HLA-B*57:01-LF9 (Protein Data Bank [PDB] accession code 3VH8). The search model used for the binary complexes was the previously determined structure of HLA-B*57:01 with the peptide removed (PDB accession code 3VH8) (29). Model refinement was carried out in PHENIX (32) with iterative rounds of manual building in COOT (33). Solvent molecules were added with COOT, and the structures were validated with MolProbity (34). The final refinement values are summarized in Table 2. The KIR3DS1-HLA-B*57:01-LF9 ternary complex was modeled on the KIR3DL1*001-HLA-B*57:01-LF9 crystal structure (PDB accession code 3VH8) (12). The substitutions I47V, S58G, V92M, G138W, P163S, L166R, and P198L were generated and manually fitted in COOT (33). The model was energetically minimized with geometry restraints in REFMAC (35).

### Statistical analysis.

One-way analysis of variance (ANOVA) with Dunnett's multiple comparison posttest was performed using GraphPad Prism, version 5.0a, for Mac OS X (GraphPad Software, San Diego, CA, USA).

## RESULTS

### KIR3DL1 recognition is influenced by HLA-B*57:01-LF9 peptide variants.

It is established that C-terminal peptide residues in the pHLA complex critically impact KIR3DL1 recognition (12, 16, 36, 37). For example, in the context of HLA-B*57:01 bound to LF9 (LSSPVTKSF), replacing the serine (S) at position 8 (P8) with the negatively charged residue glutamate (E) or the smaller, nonpolar residue alanine (A) dramatically reduced binding to KIR3DL1 (12). Such peptide modifications could affect KIR3DL1 binding directly by disrupting interactions with the KIR molecule and/or indirectly by altering the structure of the HLA molecule and, in particular, the critical Bw4 motif (38).

**FIG 1:**
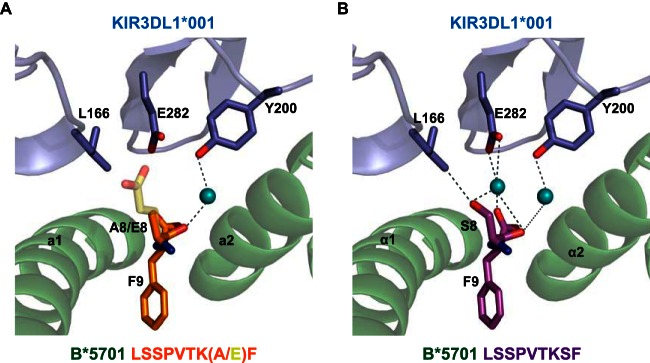
The role of peptide in KIR3DL1-HLA interactions. (A) The structures of KIR3DL1*001-HLA-B*57:01-LF9.A8 ternary complex and the HLA-B*57:01-LF9.E8 binary complex were solved to a resolution of 2.3 and 1.9 Å, respectively. (B) The structure of the KIR3DL1*001-HLA-B*57:01-LF9 ternary complex used for comparison. HLA and KIR are shown in green and blue, respectively; amino acids at position P8 and P9 are highlighted. Solid lines indicate direct contacts; dashed lines represent water-mediated contacts.

To address these possibilities, we solved the binary structure of HLA-B*57:01 in complex with LF9 E8 (Table 2) and compared this to the structure of KIR3DL1*001 in complex with HLA-B*57:01-LF9 (Fig. 1). These data showed that the side chain of glutamic acid at P8 protruded out of the peptide-binding cleft but did not significantly alter the Bw4 epitope. However, modeling the ternary interaction revealed both steric and charge-mediated clashes with KIR3DL1*001, most notably via E282 in the KIR D2 domain (Fig. 1A).

In contrast to LF9 E8, the sensitivity of KIR3DL1*001 to alanine substitution at P8 was more puzzling. To explore this observation further, we determined the ternary structure of KIR3DL1*001-HLA-B*57:01-LF9.A8 (Fig. 1A and Table 2). This mutant complex could be superimposed closely on the corresponding wild-type ternary structure, and, again, no conformational adjustments of the Bw4 motif were observed. However, the alanine residue at P8 was positioned such that it could no longer contact L166 in the KIR D1 domain. Accordingly, this amino acid substitution abolishes the only direct peptide interaction with KIR3DL1*001.

### Nonpermissive role for KIR3DS1-like residues is dependent on both HLA and peptide.

**FIG 2:**
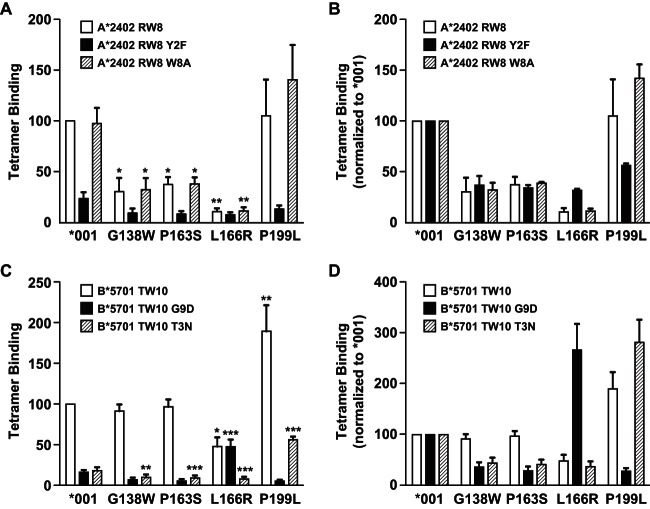
The nonpermissive role of KIR3DS1-like residues is dependent on both HLA and peptide. (A to D) HEK293T cells were transfected with FLAG-tagged KIR3DL1*001 or single amino acid variants thereof and stained with individual pHLA class I tetramers as indicated. Results are expressed relative to HLA-A*24:02-RW8 (A) or HLA-B*57:01-TW10 (C) binding to KIR3DL1*001 or relative to the binding of each tetramer to KIR3DL1*001 (B and D). Data are averaged from three independent experiments. Error bars represent standard errors of the means. Binding of each tetramer was compared to that seen with KIR3DL1*001 using a one-way ANOVA, followed by a Dunnett's multiple comparison posttest. *, *P* < 0.05; **, *P* < 0.01; ***, *P* < 0.001.

KIR3DS1 contains a cluster of amino acid substitutions not typically found in KIR3DL1 (W138, S163, R166, and L199) that can limit HLA recognition (24, 25). We used a panel of tetramers of HLA allotypes presenting a variety of peptides to examine the role of these residues in the context of KIR3DL1*001. The presence of W138, S163, or R166, but not L199, strongly inhibited binding to HLA-A*2402 (Fig. 2A and B), whereas only the R166 mutation impaired binding to HLA-B*57:01 (Fig. 2C and D). In addition to these allotype-specific differences, peptide-dependent effects further modulated the binding sensitivity of KIR3DS1-like mutants. For example, binding of HLA-A*24:02 refolded with the HIV-derived peptide RW8 (RYPLTFGW; Nef, residues 134 to 141) was unaffected by the L199 mutation. In contrast, an appreciable loss of binding to KIR3DL1*001 was observed with the variant peptide RW8 Y2F, compounded in the presence of L199, which was not apparent with the variant peptide RW8 W8A. Similarly, binding of HLA-B*57:01 refolded with the HIV-derived peptide variant TW10 G9D (TSTLQEQIDW; Gag p24, residues 108 to 117) was substantially reduced relative to that of the wild type. However, the R166 mutant form of KIR3DL1*001 displayed a clear preference for this variant peptide complexed with HLA-B*57:01, and binding was significantly higher than that seen with KIR3DL1*001. The role of KIR3DS1-like residues in ligand engagement is therefore influenced by both components of the pHLA complex.

### KIR3DS1-like residues interact to influence peptide specificity.

**FIG 3:**
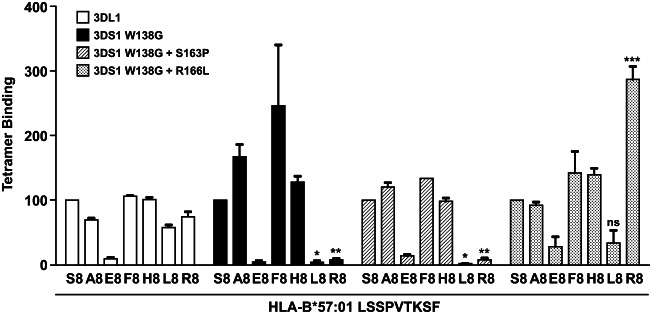
KIR3DS1-specific residues influence peptide specificity. HEK293T cells were transfected with FLAG-tagged KIR3DL1*001 or amino acid variants of KIR3DS1 and stained with individual HLA-B*57:01-LF9 tetramers incorporating bound peptide substitutions at P8, as indicated. Results are expressed relative to HLA-B*57:01-LF9 binding for each KIR construct. Data are averaged from three independent experiments. Error bars represent standard errors of the means. Binding of L8 and R8 variants of peptide LF9 to each mutant was compared to that seen with KIR3DL1*001 using a one-way ANOVA, followed by a Dunnett's multiple comparison posttest. *, *P* < 0.05; **, *P* < 0.01; ***, *P* < 0.001.

In a complementary set of experiments, we assessed the role of peptide in HLA binding to modified KIR3DS1. Previously, we showed that HLA engagement required only a single mutation (W138G) in KIR3DS1 (25); this binding was not seen with other single reversion mutations including those at position 166. However, the pattern of peptide reactivity was noticeably different between this KIR3DS1 variant (3DS1*014) and KIR3DL1 (Fig. 3). Introduction of a second KIR3DL1-like residue (S163P or R166L) also modified the pattern of reactivity. For example, KIR3DL1 bound moderately to HLA-B*57:01 complexed with LF9 R8, whereas KIR3DS1 W138G showed no such binding. In contrast, the further introduction of R166L generated a KIR3DS1 molecule that engaged HLA-B*57:01-LF9.R8 robustly. These data suggest that the KIR3DS1-like residues interact in a complex fashion to impair HLA affinity and modify peptide preferences.

### Arginine at position 166 in KIR3DS1 limits HLA interaction.

**FIG 4:**
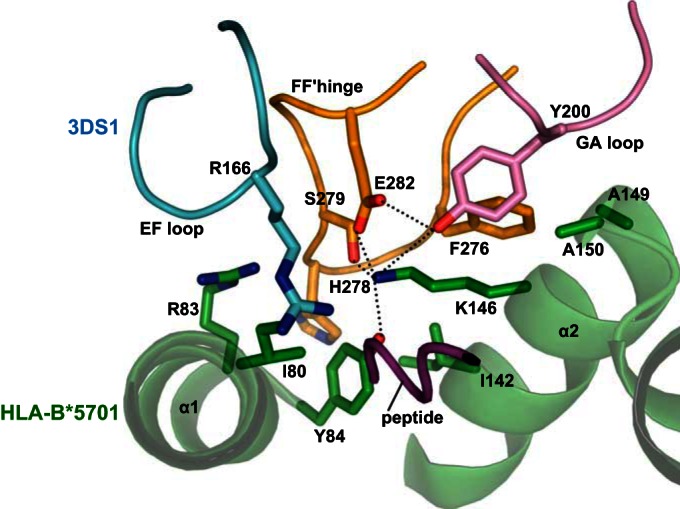
Modeling of KIR3DS1 residues suggests a steric clash between R166 and HLA-Bw4 R83. Using the structure of KIR3DL1*001-HLA-B*57:01-LF9 for reference, the KIR3DS1-like R166 residue was modeled in place of the KIR3DL1 residue L166. HLA and peptide are shown in green and purple, respectively. KIR is shown in light blue (D1 residues), orange (D2 residues), and pink (D1-D2 loop). The presence of R166 in KIR3DS1 is predicted to cause both a steric and a charge clash with R83 in HLA-B57:01.

To further understand the role of KIR3DS1-like residues, both individually and in concert, we modeled the interaction of KIR3DS1 with HLA-B*57:01 based on the structure of KIR3DL1 (Fig. 4). From this model, it emerged that HLA binding is primarily impeded by the presence of R166 in KIR3DS1. *In silico*, this substitution causes a steric and charge-mediated clash with R83 in HLA-B*57:01. The characteristics of this model are consistent with our finding that the L166R substitution in KIR3DL1 results in a loss of affinity for a broad range of HLA-Bw4 allotypes (Fig. 2).

### Peptide-dependent binding of HLA-B*57:01 to KIR3DS1.

**FIG 5:**
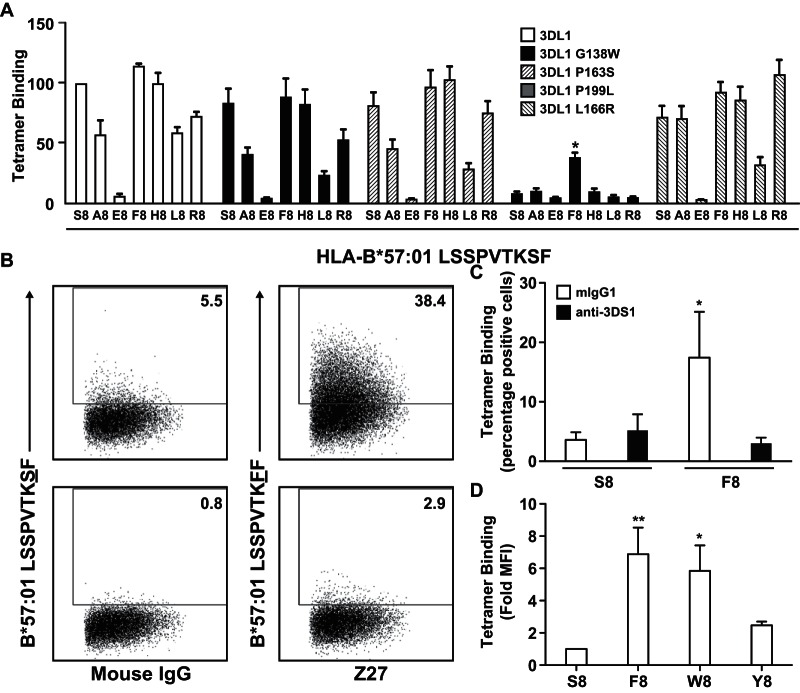
The interaction between HLA-B*57:01 and KIR3DS1 is peptide dependent. (A) HEK293T cells were transfected with FLAG-tagged KIR3DL1*001 or single amino acid variants thereof and stained with individual HLA-B*57:01-LF9 tetramers incorporating bound peptide substitutions at P8, as indicated. Data are averaged from three independent experiments. Error bars represent standard errors of the means. For KIR3DL1*001 L166R, binding of each peptide variant was compared to that seen with HLA-B*57:01 LF9 using a one-way ANOVA followed by a Dunnett's multiple comparison posttest. (B) HEK293 cells were transfected with FLAG-tagged KIR3DS1 and stained with either HLA-B*57:01-LF9 or HLA-B*57:01-LF9.F8 tetramer in the presence of control mouse IgG or the blocking antibody Z27. The frequency of positive cells is depicted in each gate. (C) Data averaged from three independent experiments as shown in panel B. Binding of each peptide variant was compared to that of LF9 (S8) in the presence of control mouse antibody using a one-way ANOVA followed by a Dunnett's multiple comparison posttest. (D) HEK293 cells were transfected with FLAG-tagged KIR3DS1 and stained with individual HLA-B*57:01-LF9 tetramers incorporating bound peptide substitutions at P8 as indicated. MFI, median fluorescence intensity. Data are averaged from three independent experiments. Error bars represent standard errors of the means. Binding of each peptide variant was compared to that of LF9 (S8) using a one-way ANOVA followed by a Dunnett's multiple comparison posttest. *, *P* < 0.05; ** *P* < 0.01.

As the presence of arginine at position 83 is invariant in all HLA-Bw4 allotypes, the KIR3DS1 R166/HLA R83 clash would be predicted to limit the interaction of KIR3DS1 with all of these molecules. However, the presented peptide could potentially induce conformational changes at the HLA interface and impact KIR binding. To address this possibility, we scanned KIR3DL1 mutants incorporating KIR3DS1-like residues with a panel of LF9 peptides with substitutions at P8. As previously documented, KIR3DL1*001 binding was highly sensitive to P8 residue changes in the HLA-B*57:01-LF9 complex (12, 37) (Fig. 5A). Overall, the same pattern of peptide reactivity was observed with the W138, S163, and L199 mutant forms of KIR3DL1*001. In contrast, the R166 mutant interacted substantially with HLA-B*57:01-LF9.F8 despite barely recognizing the corresponding wild-type complex. These findings suggest that specific substitutions at P8 might allow KIR3DS1 to bind HLA-B*57:01. Consistent with this idea, KIR3DS1-expressing cells bound tetrameric complexes of HLA-B*57:01-LF9.F8 but not HLA-B*57:01-LF9. Moreover, this interaction was abrogated in the presence of the anti-KIR3DS1 blocking antibody Z27 (Fig. 5B and C). The epitope recognized by the Z27 antibody overlaps the HLA-binding face and prevents interaction of KIR3DL1 with its HLA ligand (data not shown).

The binding of HLA-B*57:01-LF9.F8 to KIR3DS1 suggested that other peptide residues might also be capable of supporting this interaction. Similar to phenylalanine (F), both tyrosine (Y) and tryptophan (W) contain an aromatic ring, prompting us to examine the binding of KIR3DS1 to HLA-B*57:01-LF9.W8 and HLA-B*57:01-LF9.Y8. Weak binding was observed with both of these variant pHLA complexes (Fig. 5D) although this was not statistically significant in the case of Y8, and specificity was again confirmed by blocking with Z27 (data not shown).

### Identification of pathogen-derived peptides that facilitate HLA binding to KIR3DS1.

**FIG 6:**
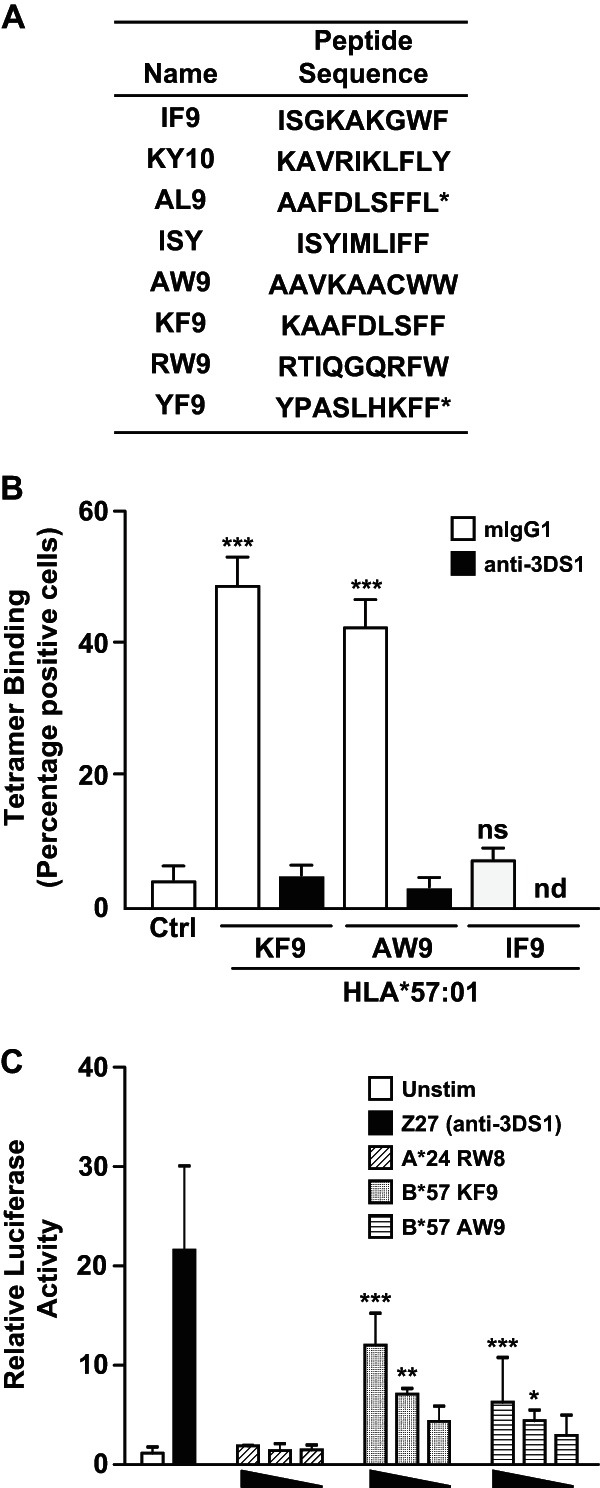
Identification of pathogen-derived peptides permissive for KIR3DS1 activation. (A) List of HLA-B*57:01-restricted peptides selected based on the presence of F or W at position PΩ-1. Peptide sequences marked with an asterisk stabilized HLA-B*57:01 poorly *in vitro* and were not used in subsequent binding experiments. (B) Mock- or FLAG-tagged KIR3DS1-transfected HEK293T cells were stained with individual HLA-B*57:01 tetramers as indicated in the presence of control mouse IgG or the blocking antibody Z27. Data are averaged from three independent experiments. Error bars represent standard errors of the means. Binding was compared to unstained control using a one-way ANOVA followed by a Dunnett's multiple comparison posttest. (C) Decreasing concentrations of plate-bound pHLA monomer were used to stimulate KIR3DS1-CD3ζ Jurkat cells. NFAT activity was measured using a luciferase reporter and normalized to constitutive TK-driven Renilla activity. Data are averaged from three independent experiments. Error bars represent standard errors of the means. Activation was compared to that of unstimulated controls using a one-way ANOVA followed by a Dunnett's multiple comparison posttest. *, *P* < 0.05; **, *P* < 0.01; ***, *P* < 0.001; ns, not significant; nd, not done.

The data presented above suggest that, in the context of HLA-B*57:01, the presence of certain residues at P8 (F, Y, and W) can facilitate recognition by KIR3DS1. To extend these findings to a pathogen-relevant context, we explored peptide databases including the Immune Epitope Database and Analysis Resource (http://www.iedb.org), SYFPEITHI (http://www.syfpeithi.de/), MHCBN (http://www.imtech.res.in/raghava/mhcbn/index.html), and the Los Alamos National Laboratory HIV database (http://www.hiv.lanl.gov/content/immunology/). In particular, we searched for confirmed peptide epitopes that were either known to bind HLA-B*57 allotypes or fit the consensus sequence for binding to HLA-B*57:01 (P2, A/T/S; PΩ-1, W/F [where PΩ is the C-terminal anchor position]) (39) that also met our criteria for binding to KIR3DS1 (P8/PΩ-2, F/W/Y) (Fig. 5). Based on these specifications, we generated a short list of eight peptides for further analysis (Fig. 6A). The corresponding pHLA-B*57:01 tetramers were generated and tested against both LILRB1 and KIR3DS1. Two of the pHLA-B*57:01 complexes refolded poorly, likely due to low-affinity peptide loading. Of the six remaining tetramers, two interacted specifically with KIR3DS1: (i) AW9 (AAVKAACWW; HIV Pol, residues 839 to 847) and (ii) KF9 (KAAFDLSFF; HIV Nef, residues 82 to 90). In both cases, binding was completely inhibited in the presence of Z27 (Fig. 6B).

### Activation of KIR3DS1 by pHLA complexes.

To determine whether these pHLA-B*57:01 complexes could generate an activation signal via KIR3DS1, we used a chimeric KIR3DS1-CD3ζ Jurkat cell line. These cells were stimulated with plate-bound pHLA-B*57:01 complexes, and NFAT activity was measured using a luciferase reporter construct. Cross-linking of KIR3DS1 with the Z27 antibody resulted in robust luciferase activity. Dose-dependent stimulation was also observed with both HLA-B*57:01-KF9 and HLA-B*57:01-AW9 but not with the control HLA-A*24:02-RW8 complex (Fig. 6C). These data therefore provide the first direct evidence of functionally relevant peptide-dependent HLA-B*57:01 binding to KIR3DS1.

## DISCUSSION

Numerous studies have associated the presence of particular KIR genes and KIR/HLA gene combinations with clinical outcomes in the face of various immune challenges. Although much research in this area has focused on the ability of KIRs to detect HLA class I downregulation, also known as “missing self,” the presented peptide repertoire can also perturb KIR-HLA interactions and allow for more specific NK cell responses to “altered self.”

To establish the role of KIR residues in the phenomenon of peptide selectivity, we explored the interaction of KIR3DL1 with HLA-Bw4 using a structure/function-based approach. In line with previous evidence, KIR3DL1 binding was critically determined by amino acid residues at the peptide C terminus. Furthermore, our data suggest that this effect is governed directly by the ability of specific C-terminal residues to interact productively with KIR residues rather than indirectly via conformational changes in the Bw4 epitope. These limitations are imposed by at least two different physicochemical characteristics of the relevant peptide residues, namely, charge and size.

The loss of binding in complexes with small amino acid residues at P8 may reflect an inability to contact leucine at position 166 in the KIR3DL1 D1 domain. Targeting of this KIR residue had little effect on the interaction with pHLA, however, suggesting that additional factors contribute to the observed loss of binding. In this regard, it is notable that the presence of a small amino acid at this position in the peptide also results in the generation of a cavity within the structural interface. Such cavities are known to reduce protein stability (40), which in turn could potentially impact binding affinity. In contrast, our findings suggest that negatively charged residues, as present in the LF9 E8 and TW10 D9 peptides, dramatically disrupt interactions with KIR3DL1. These results are in line with a previous study (41), which reported that glutamic acid at P8 impeded KIR3DL1 binding to HLA-B*27:05. Similarly, another study found that glutamic acid and aspartic acid at P8 negatively impacted KIR3DL1 binding to two different pHLA-B*57:03 complexes (36). Our data further suggest that this intolerance of negatively charged residues relates directly to the E282 residue present in KIR3DL1. This amino acid lies centrally in the interaction zone between KIR3DL1 and HLA-B*57:01, and mutation to the similarly sized but neutral glutamine residue profoundly decreases binding (12). However, perturbation of E282 can also result in unique peptide specificity profiles (37), suggesting additional roles in peptide discrimination beyond the potential for electrostatic clashes.

Although KIR3DS1 displays a very high degree of similarity to KIR3DL1 in the extracellular domain, the HLA binding characteristics of this activating receptor are dramatically different. Our data show that KIR3DS1-specific residues interact to determine HLA restriction and peptide specificity. One KIR3DS1 residue in particular (R166) plays a dominant role in the observed lack of binding to a broad array of pHLA complexes. Molecular modeling is consistent with these biochemical findings and predicts a significant steric and electrostatic clash in hypothetical KIR3DS1-HLA-Bw4 complexes. Nonetheless, certain peptides can overcome the effects of this arginine residue in both KIR3DL1 L166R mutants and wild-type KIR3DS1 molecules. In particular, peptides with large aromatic residues at P8 allowed weak, but highly reproducible, binding to KIR3DS1 with sufficient avidity to deliver an activation signal. In contrast to phenylalanine, tryptophan, and, to a lesser extent, tyrosine, the presence of histidine at position 8 did not facilitate KIR3DS1 binding. This may be due to subtle differences in the size and orientation of His relative to the size and orientation of other aromatic amino acids. In addition, at physiological pH, some portion of His residues will be charged, which, in light of the inhibitory effect of charged peptide residues in KIR3DL1 interaction, is predicted to disrupt binding. It remains unclear how the presence of permissive peptide residues overcomes the negative impact of arginine at position 166. One possibility is that the aromatic rings of these residues stack well with arginine side chains to promote productive interactions (42), while their large size prevents the formation of any destabilizing cavities at the interface. It is also notable that the rare KIR3DS1*014 allotype, which incorporates one amino acid change (W138G) but retains R166, exhibits robust HLA recognition (25). Collectively, these observations suggest that the normally disruptive effects of R166 can be circumvented by changes at the KIR3DS1-HLA-Bw4 interface, occurring either due to the presence of certain HLA-bound peptides or as a consequence of mutations at neighboring positions in the KIR3DS1 molecule.

Our findings suggest that the multiple changes in KIR3DS1 are driven to restrict, but not abolish, the capacity of this receptor to interact with HLA-Bw4. Peptide-specific activation of KIR3DS1 may therefore only occur when the array of potential epitopes displayed on a target cell is appropriately altered in the context of infection, stress, or transformation. Viral infections, including HIV infection, are known to result in substantial changes in peptide repertoire due to the presence of peptides from virally derived proteins, stress-induced proteins (43), and peptides produced by the immunoproteasome (44). Thus, this may be a general mechanism for the engagement of both activating and inhibitory KIR to be modulated in response to viral infection. Consistent with this notion, we identified two HIV-derived peptides that enable HLA-B*57:01 to interact with KIR3DS1. Although not dependent on peptide, in the murine system Ly49H binding to the murine CMV (MCMV)-derived m157 protein sets a precedent for such pathogen-dependent activation (45, 46). Notably, this interaction leads to an expansion of Ly49H^+^ NK cells, as has been reported for the KIR3DS1^+^ NK cell subset in HCMV and HIV infection (9, 47).

Although genetic disease association studies suggest a role for activating KIRs in the outcome of HIV infection and although functional analyses have enhanced our understanding of KIR3DS1^+^ NK cell responses (8, 9), the lack of identifiable HLA-Bw4 ligands continues to limit mechanistic insights into the biology of these receptors during the disease process. Our work demonstrates for the first time that KIR3DS1 can interact productively with HLA-Bw4 in the context of HIV infection. By extension, these data support a model whereby changes in the peptide repertoire associated with viral infection provide a trigger for KIR3DS1 engagement and NK cell activation.
